# Glucolipid metabolic disorders and ferroptosis in diabetic ulcers: pathogenic crossroads and therapeutic opportunities

**DOI:** 10.3389/fendo.2026.1760943

**Published:** 2026-01-28

**Authors:** Zaiying Yeerbao, Xinxi Li, Lei Zhang, Guoli Du, Jingdong Tang, Shuai Jiang, Xiangxiang Ru, Donglin Li, Dilinuerkezi Abulimiti, Li Chen, Yuxin Deng, Guliteken Aihemaitijiang, Halizhati Halimulati, Yunshan Li, Ye Tian

**Affiliations:** 1 Department of Vascular and Thyroid Surgery, First Affiliated Hospital of Xinjiang Medical University, Urumqi, China; 2 Department of Endocrinology, First Affiliated Hospital of Xinjiang Medical University, Urumqi, China; 3 Department of Vascular Surgery, Shanghai Pudong Hospital, Fudan University, Shanghai, China

**Keywords:** ACSL4/Nrf2/AGEs-RAGE axis, diabetic ulcers, DUs, GLMDs, glucolipid metabolic disorders

## Abstract

Ferroptosis, an iron-dependent form of regulated cell death driven by lipid peroxidation, has emerged as a key player in the pathogenesis of Diabetic Ulcers (DUs). Concurrently, Glucolipid Metabolic Disorders (GLMDs), driven by persistent hyperglycemia and lipotoxicity, constitute a core pathological basis for tissue damage and dysfunction of reparative cells. The interplay between ferroptosis and GLMDs represents a promising yet underexplored research area. This review synthesizes recent advances in understanding the molecular mechanisms underlying their interaction, focusing on how GLMDs drive ferroptosis by supplying lipid peroxidation substrates, inducing iron overload, and weakening antioxidant defenses. It also elaborates on how ferroptosis, in turn, exacerbates local metabolic stress and chronic inflammation through the release of Damage-Associated Molecular Patterns (DAMPs), thereby creating a vicious cycle. We propose that the ACSL4/Nrf2/AGEs-RAGE axis acts as a critical molecular hub integrating upstream metabolic insults with downstream ferroptotic execution, representing a novel and targetable pathogenic circuit in DUs. Modulating key molecules within this axis (e.g., ACSL4, GPX4, Nrf2) offers promising therapeutic strategies for breaking this cycle, either by selectively eliminating harmful cells or protecting reparative ones. This review aims to bridge current knowledge gaps and provide a mechanistic foundation for developing innovative therapies that combine metabolic regulation with ferroptosis intervention.

## 1 Introduction

Diabetic ulcers (DUs) represent a severe and prevalent complication of diabetes, characterized by impaired healing, high risk of infection, and substantial morbidity (1, 2).The pathogenesis of DUs is multifaceted, involving peripheral neuropathy, ischemia, and a persistent pro-inflammatory microenvironment. Beyond these established factors, dysregulated cellular demise and profound systemic metabolic disturbances are now recognized as central culprits in driving the non-healing phenotype. The pathogenesis of diabetic foot ulcers is multifactorial and traditionally attributed to a triad of peripheral neuropathy, ischemia from peripheral arterial disease, and susceptibility to infection. Neuropathy leads to loss of protective sensation and foot deformity, setting the stage for unrecognized injury (3). Ischemia impairs tissue perfusion and oxygen delivery, critically undermining the repair process. In this vulnerable environment, even minor wounds can escalate into chronic, non-healing ulcers prone to infection. While this triad explains the initiation and perpetuation of ulcers at a macroscopic level, it provides less insight into the persistent cellular and molecular dysfunction within the wound microenvironment that defies standard therapeutic interventions. A critical gap exists in understanding the dynamic crosstalk between systemic metabolic dysregulation and dysregulated cell fate at the ulcer site. Furthermore, diabetic neuropathy and nephropathy are strongly associated with an increased risk of major adverse foot events, underscoring the severe clinical progression of DU (4). Among these, the interplay between ferroptosis—a novel iron-dependent cell death—and glucolipid metabolic disorders (GLMDs) (5) has recently emerged as a critical yet underexplored axis in DU pathophysiology. Ferroptosis is driven by iron-catalyzed lipid peroxidation, leading to membrane damage and cell death. GLMDs, encompassing chronic hyperglycemia and lipotoxicity, create a hostile metabolic milieu that disrupts tissue homeostasis. Crucially, emerging evidence suggests that in DUs, ferroptosis and GLMDs are not parallel pathways but engage in a destructive bidirectional crosstalk, forming a self-amplifying vicious cycle that perpetuates tissue damage and impedes repair. For instance, disordered glucose metabolism resulting from chronic hyperglycemia in diabetes can impair iron metabolism pathways, significantly reducing the iron-binding capacity of ferritin, leading to increased plasma free iron, and subsequently promoting lipid peroxidation and ferroptosis (6).Furthermore, glucose has been demonstrated to be necessary for ferroptosis, while glucose metabolism disorders at low glucose levels can prevent ferroptosis (7).Similarly, in pancreatic ductal adenocarcinoma cells within a metabolic disease context, high glucose was found to promote glycolysis, enhance pyruvate oxidation, stimulate the tricarboxylic acid cycle and fatty acid synthesis, ultimately promoting lipid peroxidation-dependent ferroptosis (8).ron, a key cofactor for lipid peroxidation, is stringently regulated by both glucose and lipid metabolism. GLMDs play a crucial role in modulating iron metabolism and lipid peroxidation. This intricate interaction positions the ferroptosis-GLMD axis as a pivotal frontier for understanding DU pathogenesis and uncovering new therapeutic targets.

On one hand, GLMDs predispose cells to ferroptosis by supplying abundant peroxidizable lipid substrates, disrupting iron homeostasis, and compromising key antioxidant defenses. On the other hand, cells undergoing ferroptosis release damage signals that exacerbate local inflammation and metabolic stress, thereby worsening GLMDs. This review aims to comprehensively synthesize current knowledge on this dynamic interplay. We will first outline the core mechanisms of ferroptosis and GLMDs, then delve into their molecular interactions within the DU microenvironment, with a focus on key hubs such as the Acyl-CoA Synthetase Long-Chain Family Member 4 (ACSL4), Nuclear factor erythroid 2-related factor 2 (Nrf2), and the Advanced Glycation End products-Receptor for Advanced Glycation End products (AGEs-RAGE) pathways. Finally, we will discuss the translational potential of targeting this axis and propose future research directions to advance DU therapy.

## 2 Basic concepts and molecular mechanisms of ferroptosis

### 2.1 The concept of ferroptosis and comparison with other cell death mechanisms

Ferroptosis is a recently proposed concept of cell death driven by iron-dependent lipid peroxidation. It was first identified by Dr. Brent R. Stockwell in 2012. This unique cell death mode occurs when the reduction reaction catalyzed by glutathione peroxidase 4 (GPX4) fails to eliminate excessive lipid peroxides. Oxidative damage disrupts systemic iron homeostasis, leading to massive inactivation of intracellular glutathione (GSH) and GPX4, resulting in ferroptosis (9).Compared to other cell death types, ferroptosis exhibits distinctive morphological changes, such as shrunken and condensed mitochondria (10), Apoptosis was once considered the sole regulated cell death pathway; in mammalian cells, it can be classified into intrinsic and extrinsic apoptosis. Extrinsic apoptosis is mediated by the activation of death receptors (e.g., TNFR1) located on the plasma membrane, while intrinsic apoptosis can be activated following cellular alterations like DNA damage or mitochondrial injury (11).In contrast, necroptosis may have evolved as a lytic cell death mode serving as an antiviral defense mechanism. Apoptotic signaling is transduced via caspase-8 proteases, and when caspase-8 is inhibited, necroptosis may occur (12), characterized by cell swelling, loss of membrane integrity, and leakage of cellular contents into the surrounding tissue. Pyroptosis, a novel form of programmed cell death mediated by gasdermin (GSDM) family members, is characterized by cell swelling, plasma membrane rupture, and release of inflammatory mediators like interleukin-1β (IL-1β) and IL-18 (13).Recent studies on diabetic retinopathy also found the NLRP3 inflammasome activated by various pathways (e.g., ATP), leading to IL-1β and IL-18 secretion, pyroptosis, and accelerated disease progression (14).In contrast, ferroptosis is morphologically unique, with cells exhibiting mitochondrial atrophy and condensation, representing a non-apoptotic cell death mechanism (15), Here, free intracellular iron or iron-containing enzymes react with oxygen and polyunsaturated fatty acid (PUFA)-containing lipids, generating high levels of membrane lipid peroxides that disrupt membrane integrity, causing membrane damage without complete lysis, ultimately leading to cell death. The distinctive features of ferroptosis, apoptosis, necroptosis, and pyroptosis relevant to DU pathogenesis are systematically compared in **Table 1**.

**Table 1:** A comparative overview of regulated cell death modalities in diabetic ulcer pathophysiology.

Characteristic	Ferroptosis	Apoptosis	Necrosis	Pyroptosis
Key Triggers	Iron overload, Lipid peroxidation	DNA damage, Death receptor activation	Physical/Chemical damage, ATP depletion	Inflammasome activation, Gasdermin cleavage
Key Regulators	GPX4, ACSL4, NCOA4, FSP1	Caspases, Bcl-2 family	RIPK1, RIPK3, MLKL	Caspase-1, GSDMD, NLRP3
Morphological Features	Mitochondrial shrinkage, Increased membrane density	Cell shrinkage, Chromatin condensation, Apoptotic bodies	Cell swelling, Plasma membrane rupture	Cell swelling, Plasma membrane pore formation
Immunogenicity	Moderate/High (Releases DAMPs, e.g., HMGB1)	Low (Generally immunologically silent)	High (Strongly pro-inflammatory)	High (Releases IL-1β, IL-18)
Role in DU	Dual role: Clearance of senescent fibroblasts (16) vs. injury to reparative cells (e.g., endothelial cells) (17, 18)	Often impaired, leading to survival of damaged cells (19)	Exacerbates tissue damage and inflammation (20)	Promotes chronic inflammation, impedes healing (14, 21)
Therapeutic Implications	Induce (to eliminate harmful cells) or Inhibit (to protect reparative cells)	Overcome apoptosis resistance	Inhibit necroinflammation	Inhibit pyroptosis to mitigate inflammation

ACSL4, Acyl-CoA synthetase long-chain family member 4; DAMPs, Damage-associated molecular patterns; DU, Diabetic ulcer; FSP1, Ferroptosis suppressor protein 1; GPX4, Glutathione peroxidase 4; GSDMD, Gasdermin D; IL-1β, Interleukin-1β; IL-18, Interleukin-18; MLKL, Mixed lineage kinase domain-like pseudokinase; NCOA4, Nuclear receptor coactivator 4; NLRP3, NOD-, LRR- and pyrin domain-containing protein 3; PAMPs, Pathogen-associated molecular patterns; RIPK1, Receptor-interacting serine/threonine-protein kinase 1; RIPK3, Receptor-interacting serine/threonine-protein kinase 3; TNF-α, Tumor necrosis factor-alpha.

### 2.2 Key molecules and regulatory pathways

**Figure 1:**
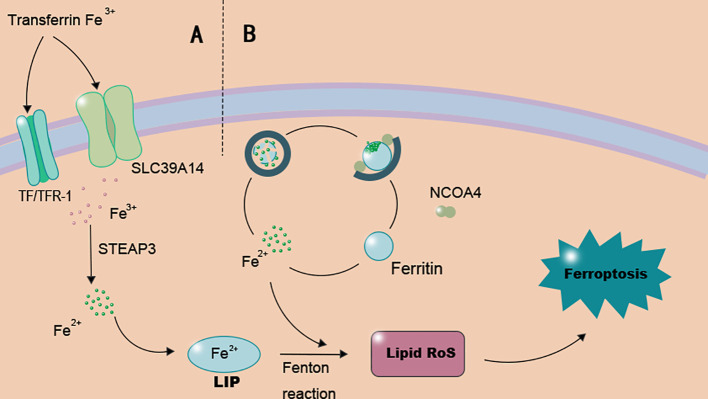
Key molecular pathways inducing ferroptosis. The graphic illustrates the core mechanisms that drive ferroptosis initiation, primarily involving iron overload and lipid peroxidation. **(A)** Iron metabolism dysregulation: Cellular iron uptake via transferrin receptor 1 (TFR1) and subsequent reduction lead to an expanded labile iron pool (LIP). Excess Fe^2+^ fuels the Fenton reaction, generating reactive oxygen species (ROS) that initiate lipid peroxidation. **(B)** Ferritinophagy: NCOA4-mediated autophagic degradation of ferritin releases stored iron, further amplifying the LIP and cellular susceptibility to ferroptosis. The accumulation of lipid peroxides on cellular membranes, particularly those rich in phosphatidylethanolamine (PE) esterified with polyunsaturated fatty acids (PUFAs) by ACSL4 and LPCAT3, culminates in membrane damage and ferroptotic cell death.

Ferroptosis is controlled by numerous interconnected signaling pathways. This complex regulatory network underscores its crucial role in cellular homeostasis and disease progression. The regulation of ferroptosis involves a sophisticated molecular network, including GPX4, Reactive Oxygen Species (ROS), and a suite of lipid peroxidation-regulating enzymes. Various pathways can modulate the initiation and progression of ferroptosis, primarily through dysregulated iron metabolism and enhanced lipid peroxidation as summarized in **Figure 1**.

#### 2.2.1 Pathways inducing ferroptosis

##### 2.2.1.1 Iron metabolism dysregulation and the Fenton reaction

Ferroptosis occurs through the accumulation of intracellular ferrous iron (Fe^2+^) and lipid peroxides, leading to membrane damage and eventual cell death. Iron enters cells via the transferrin/transferrin receptor (TF/TFR-1) transport system or the SLC39A14 channel (22), is reduced to Fe^2+^ by metalloreductases (e.g., STEAP3), and subsequently transported into the cytosolic labile iron pool (LIP) via DMT1 or iron metabolism regulator proteins (ZIP8/14) (23). Excess Fe^2+^ in the LIP can participate in the Fenton reaction, generating reactive oxygen species (ROS) that promote lipid peroxide formation. ROS accumulation induces membrane lipid peroxidation, causing loss of cellular function and cell death (as illustrated in **Figure 1A**). Importantly, both cellular iron uptake and the redox state of the labile iron pool are susceptible to modulation by the diabetic metabolic milieu, directly linking systemic glucose and lipid disorders to this core ferroptotic trigger.”

Dysregulated iron metabolism is crucial for modulating ferroptosis, particularly in controlling the size of the LIP. Intracellular free iron plays vital roles in many cellular processes, including the production of iron-containing proteins and the storage of excess iron in ferritin, both regulated by Iron Regulatory Proteins (IRPs). The LIP modulates the expression of genes involved in iron metabolism through post-transcriptional regulation by IRPs, thereby fine-tuning the levels of iron metabolism-related proteins (24).

##### 2.2.1.2 Ferritinophagy

Ferritinophagy, the autophagic degradation of ferritin, represents another critical source of catalytic iron for ferroptosis. This process is mediated by Nuclear Receptor Coactivator 4 (NCOA4), which directs ferritin to lysosomes for degradation, thereby releasing free iron and increasing the labile iron pool (25) (see the ferritinophagy pathway depicted in **Figure 1B**). In the context of DU, dysregulation of this pathway contributes to the cellular iron overload that fuels lipid peroxidation.

#### 2.2.2 Pathways inhibiting ferroptosis

**Figure 2:**
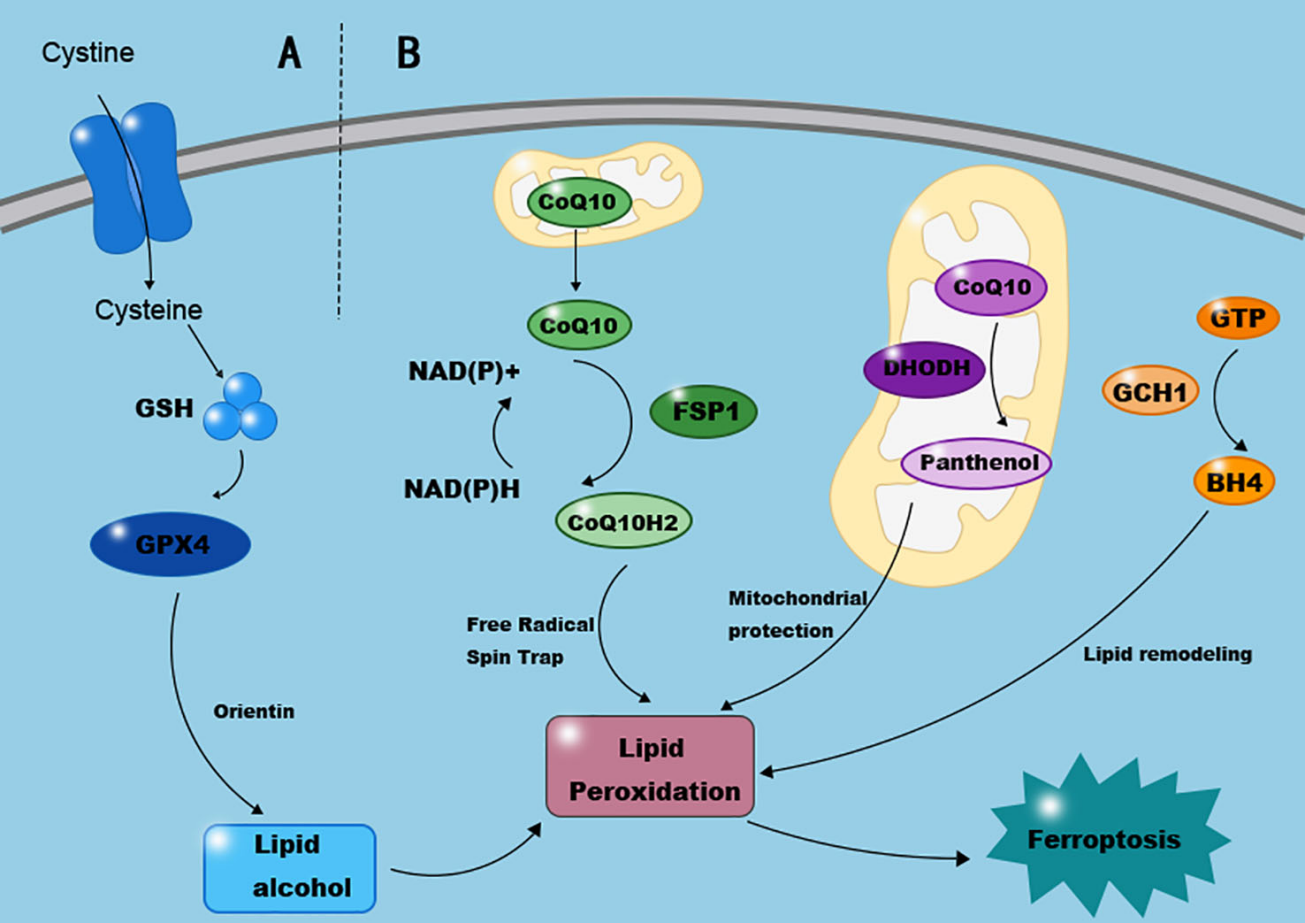
Key pathways conferring resistance to ferroptosis. This diagram summarizes the principal endogenous defense systems that protect cells against ferroptosis. **(A)** The canonical System Xc–/GSH/GPX4 axis: The cystine/glutamate antiporter (System Xc–, composed of SLC7A11 and SLC3A2) imports cystine for glutathione (GSH) synthesis. GPX4 utilizes GSH to reduce lipid hydroperoxides (L-OOH) to non-toxic lipid alcohols (L-OH), thereby preventing lethal lipid peroxidation. Compounds like Orientin and Selenium can enhance this pathway. **(B)** Non-canonical parallel pathways: The FSP1/CoQ10 system on the plasma membrane regenerates the antioxidant ubiquinol (CoQ10H2) using NAD(P)H. The mitochondrial DHODH enzyme independently produces ubiquinol within the inner mitochondrial membrane. The GCH1/BH4 pathway suppresses ferroptosis in a GPX4-independent manner by promoting lipid remodeling. Together, these systems constitute a multi-layered antioxidant network that maintains cellular redox homeostasis and inhibits ferroptosis.

Cells have evolved several mechanisms to inhibit ferroptosis, including the canonical System Xc-/GPX4 axis and parallel pathways such as FSP1/CoQ10 and DHODH, which collectively form a multi-layered antioxidant network (**Figure 2**).

##### 2.2.2.1 System Xc-/GPX4 pathway

Dysregulation of the System Xc-/GPX4 pathway is central to ferroptosis. System Xc-, composed of SLC7A11 and SLC3A2, imports cystine into the cell, which is reduced to cysteine for glutathione (GSH) synthesis. Glutathione peroxidase 4 (GPX4) utilizes GSH to reduce lipid hydroperoxides to harmless lipid alcohols, thereby neutralizing their peroxidant activity (26) (see **Figure 2A**). Failure of the GPX4-catalyzed reduction to clear excessive lipid peroxides leads to oxidative damage, disrupts iron homeostasis, inactivates intracellular GSH and GPX4, and precipitates ferroptosis. GPX4 is a key enzyme preventing ferroptosis, involved in reducing lipid peroxides to nontoxic lipid alcohols, thus protecting cells. RSL3 can directly inhibit GPX4 activity, inducing ferroptosis, as the electrophilic RSL3 binds to the nucleophilic portion of the selenocysteine residue in GPX4’s active site, impairing the glutathione cycle (6). Similarly, GPX4 reduction in T cells selectively diminished T follicular helper cells and germinal center responses in immunized mice. Selenium supplementation not only enhanced GPX4 expression in T cells and T follicular helper cell numbers but also promoted antibody responses in immunized mice and young adults after influenza vaccination (27). Regarding DUs, the natural flavonoid compound Orientin (Ori), known for its anti-inflammatory bioactivities, improved wound expression of GPX4 and angiogenesis markers upon Ori treatment, reversing diabetes-induced delayed wound healing (28). Regulating compounds like selenium and Ori can selectively activate GPX4 and inhibit ferroptosis, showing potential as therapeutic agents in endocrine and immune diseases. Crucially, the activity of this entire axis is highly dependent on cellular metabolic health, as hyperglycemia can deplete the NADPH and cysteine substrates essential for GSH synthesis, thereby rendering cells vulnerable to ferroptosis in DU.

##### 2.2.2.2 FSP1/CoQ10/NADPH pathway

Ferroptosis Suppressor Protein 1 (FSP1), an NAD(P)H-ubiquinone reductase, is the second major ferroptosis regulator discovered after GPX4. It effectively reduces vitamin K to hydroquinone, a potent antioxidant (29). This non-canonical vitamin K cycle is crucial for maintaining cell membrane structural and functional integrity, protecting cells from harmful lipid peroxidation and ferroptosis. Coenzyme Q10 (CoQ10) is a lipophilic antioxidant primarily located in the mitochondrial inner membrane. Nicotinamide adenine dinucleotide phosphate (NADPH) is a reducing coenzyme involved in numerous metabolic pathways. Recent studies indicate that FSP1 in the FSP1/CoQ10/NADPH pathway consumes NADH/NADPH to reduce coenzyme Q10 (ubiquinone) to CoQ10H2 (ubiquinol), which then traps radicals to prevent lipid peroxidation. Concurrently, it regenerates the antioxidant α-tocopherol (vitamin E), which can also be directly regenerated by FSP1 *in vitro* and captures radicals to suppress ferroptosis (30), thereby protecting cells from oxidative stress damage (corresponding to the FSP1/CoQ10 system in **Figure 2B**). In summary, the FSP1/CoQ10/NADPH pathway can regulate ferroptosis through multiple mechanisms, potentially improving patient outcomes. This discovery offers new perspectives and approaches for treating ferroptosis in non-healing DUs. The therapeutic potential of targeting this axis is highlighted by studies showing that CoQ10 analogs can ameliorate tissue injury in other disease models, such as subarachnoid hemorrhage, by specifically regulating ferroptosis and neuroinflammation (31).

##### 2.2.2.3 DHODH pathway

Dihydroorotate dehydrogenase (DHODH) is an enzyme of the mitochondrial inner membrane. Research shows that the DHODH pathway can protect cells from oxidative stress and mitochondrial damage. DHODH acts concurrently with mitochondrial GPX4 but independently of cytosolic GPX4 and FSP1 (32) (see the DHODH pathway in **Figure 2B**). Mechanistically, DHODH reduces ubiquinone to ubiquinol, an antioxidant that scavenges free radicals, thereby inhibiting ferroptosis within the mitochondrial inner membrane. The significant role of DHODH in ferroptosis within diabetic complications is supported by preclinical research; PACS2/CPT1A/DHODH signaling may function by modulating mitochondrial activity in cardiomyocytes (33), suggesting it as a novel potential therapeutic target for diabetic cardiomyopathy. While direct evidence linking DHODH to ferroptosis in DU tissues remains to be fully established, its demonstrated role in mitigating ferroptosis in other diabetic complications highlights its potential relevance within the impaired healing microenvironment of diabetic wounds, warranting further investigation.

##### 2.2.2.4 GTP cyclohydrolase 1/tetrahydrobiopterin

The GCH1/BH4 pathway involves a fundamental enzyme participating in various critical cellular metabolic processes, with GCH1 being the rate-limiting enzyme for BH4 synthesis (34). Recently, the GCH1-BH4 system has garnered considerable attention for its role in inhibiting ferroptosis. The endogenous antioxidant BH4, produced by GCH1, prevents ferroptosis in a GPX4-independent manner (35) (as shown in the GCH1/BH4 pathway, **Figure 2B**). The ability of GCH1 to increase BH4 synthesis, reduce intracellular oxidative stress, and promote cell survival makes the upregulation of GCH1 expression and subsequent elevation of BH4 levels (36), a promising therapeutic strategy for inhibiting ferroptosis in various disease contexts, including the challenge of non-healing DUs. Although the specific function of the GCH1/BH4 pathway in diabetic ulcer pathogenesis has not been extensively studied, its potent ferroptosis-suppressing activity positions it as a compelling candidate mechanism that may contribute to the regulation of redox balance and cell survival in chronic diabetic wounds.

In conclusion, understanding the mechanisms driving ferroptosis execution and resistance is decisive for developing new treatment approaches for diabetic complications, particularly DUs. By targeting the aforementioned pathways, it might be possible to achieve targeted therapy for DUs while sparing healthy patient cells, yielding more precise and effective treatments to alleviate the suffering of patients with these ulcers.

## 3 Basic concepts and molecular mechanisms of glycolipid metabolism

### 3.1 Basic concepts

**Figure 3:**
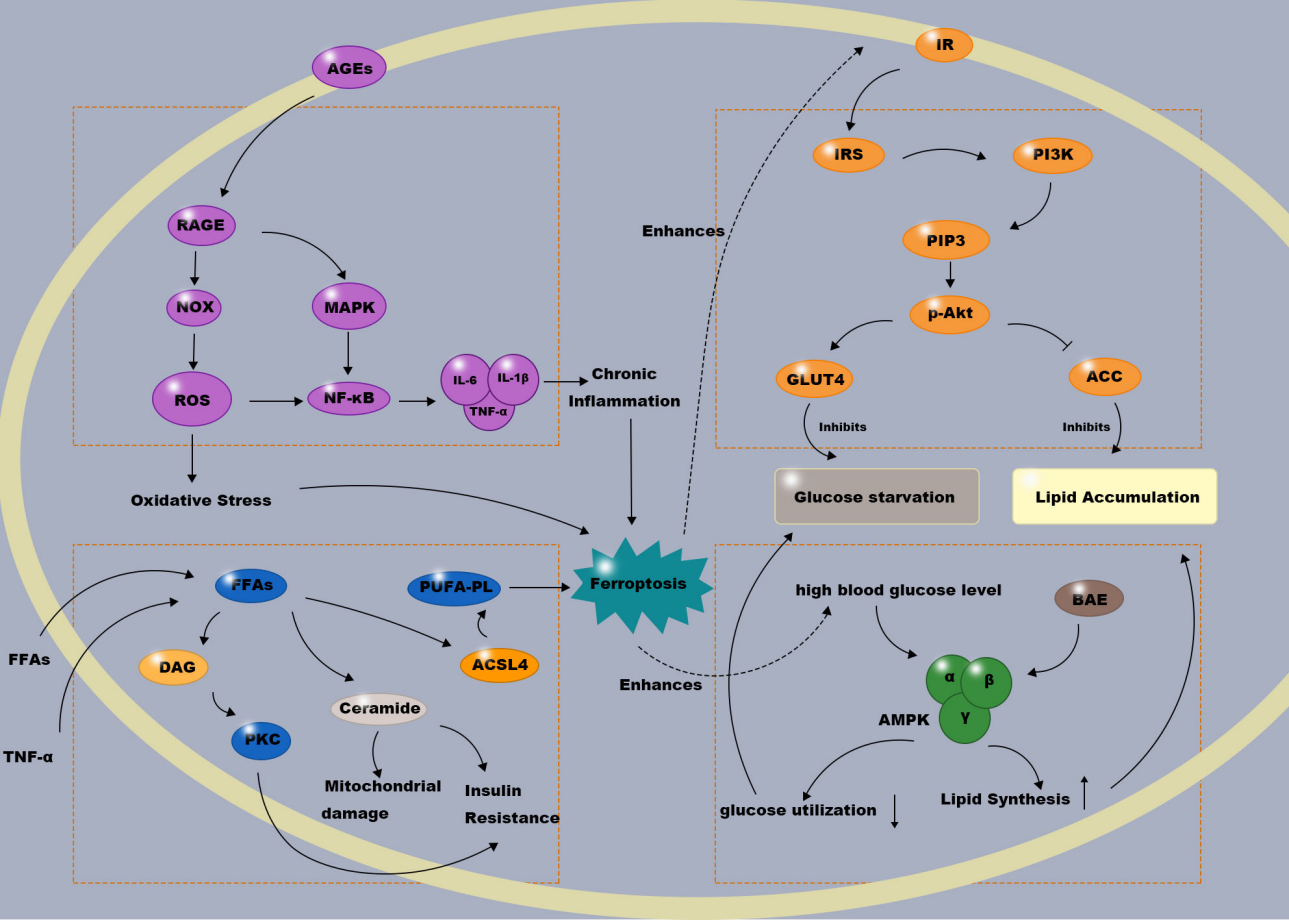
Central signaling pathways in glycolipid metabolic disorders within the diabetic ulcer microenvironment. This illustration delineates four major dysregulated pathways contributing to the metabolically toxic microenvironment that impedes diabetic ulcer healing. (1) Impaired Insulin/PI3K/Akt signaling: Hyperglycemia, free fatty acids (FFAs), and inflammation (e.g., TNF-α) induce insulin resistance via serine phosphorylation of IRS, disrupting downstream Akt activation. This leads to defective GLUT4 translocation (“cellular glucose starvation”) and unrestrained lipid synthesis. (2) Activated AGEs-RAGE axis: Persistent hyperglycemia fosters advanced glycation end products (AGEs) that engage RAGE, triggering ROS production via NOX and pro-inflammatory signaling via NF-κB. (3) Suppressed AMPK signaling: The metabolic toxicity inhibits AMPK, reducing glucose utilization and fatty acid β-oxidation while promoting lipogenesis. (4) Lipotoxicity: Elevated FFAs lead to toxic lipid species like ceramide and DAG, which exacerbate insulin resistance, mitochondrial dysfunction, and M1 macrophage polarization. These interconnected pathways collectively establish a state of energy deficit, oxidative stress, and chronic inflammation, critically undermining the healing process.

Glucose and lipid metabolism are vital processes for maintaining energy balance and normal physiological function. The molecular mechanisms of glucose metabolism involve multiple steps, including glucose uptake, glycogenesis, glycogenolysis, glycolysis, and the tricarboxylic acid (TCA) cycle. Lipid metabolism encompasses the synthesis, storage, breakdown, and utilization of lipids. Dysregulation of glucolipid metabolism contributes to the pathogenesis of various diseases. DU patients, persistently experiencing hyperglycemia and dyslipidemia, are prone to neuropathic and vascular ulcers, with GLMDs impeding ulcer healing. The complexity of GLMDs is reflected in the multitude of enzymes and proteins involved. Recent advances have identified several key signaling pathways and regulatory proteins, including macrophage polarization induced by hyperglycemia and oxidative stress (6), and the inhibition of NOX4-dependent lipid peroxidation and ferroptosis upon pharmacological intervention of the PI3K/Akt pathway (37). Hyperglycemia and abnormal lipid metabolism collectively create a “metabolically toxic microenvironment” that directly impedes various stages of wound healing (**Figure 3**).

Glycolipid metabolism plays a crucial role in DUs by providing energy and maintaining cellular function. A growing body of research finds that targeting glycolipid metabolism can effectively combat DUs (38, 39). Therefore, investigating the relationship and underlying mechanisms between glycolipid metabolism and DUs is essential for understanding the disease’s pathogenesis and identifying new therapeutic targets for its prevention and treatment.

### 3.2 Key regulatory pathways in glycolipid metabolism

#### 3.2.1 Insulin/PI3K/AKT signaling pathway

The PI3K-Akt signaling pathway is a core regulator of cell metabolism, proliferation, and survival. It plays a central role in the complex interplay between energy metabolism and cell growth (40). The insulin signaling cascade involves a series of key molecules, including the insulin receptor (IR), insulin receptor substrates (IRS), phosphatidylinositol 3-kinase (PI3K), and protein kinase B (Akt) (41), which collectively regulate a broad spectrum of genes and protein functions related to anabolism and cell survival.

Under physiological conditions, insulin binding activates a well-defined cascade through IRS, PI3K, and Akt, promoting anabolism and survival. However, in DUs, persistent hyperglycemia, free fatty acids, and inflammation induce insulin resistance. This is primarily mediated by serine phosphorylation of IRS proteins, which disrupts signal transduction (42, 43). The consequent failure to activate Akt leads to two key defects: impaired translocation of GLUT4 transporters, causing intracellular “glucose starvation” in reparative cells; and loss of inhibition on lipid synthesis, contributing to intracellular lipid accumulation (44, 45).

Consequently, in DUs, dysregulation of the insulin/PI3K-Akt pathway not only induces “glucose starvation” but also directly contributes to intracellular lipid accumulation (see the’Impaired Insulin/PI3K/Akt signaling’module in **Figure 3**).

#### 3.2.2 AGEs-RAGE signaling axis

The Advanced Glycation End products-Receptor for AGEs (AGEs-RAGE) signaling axis plays a central role in coordinating the complex interplay between metabolic disorders, chronic inflammation, and cellular dysfunction (46). The AGEs-RAGE system constitutes a complex ligand-receptor network involving various types of AGEs (e.g., CML, CEL) and their multifunctional receptor RAGE (47), which collectively trigger a cascade of downstream signaling events, leading to widespread alterations in gene expression and cellular function. Under normal conditions, AGE formation rates are low, and basal RAGE expression is limited, thus maintaining metabolic homeostasis and participating in tissue repair processes (21).

However, AGE formation is recognized as a significant pathophysiological mechanism in the development of diabetic complications. In the DU pathological milieu, this pathway is aberrantly activated. When skin tissue is chronically exposed to persistent hyperglycemia, oxidative stress, and lipid peroxidation, AGEs form abundantly and accumulate irreversibly in the extracellular matrix and circulation, inhibiting nitric oxide (NO) synthase expression in endothelial cells and impeding angiogenesis during wound healing (48). AGEs-RAGE pathway activation stimulates various downstream adaptor proteins, particularly by activating NADPH oxidases and mitogen-activated protein kinases (MAPKs), resulting in a burst of intracellular reactive oxygen species (ROS) and subsequent activation of the transcription factor NF-κB (49).

Once NF-κB is activated, its subunits (primarily p65/p50) translocate into the nucleus. There, activated NF-κB dimers bind to specific DNA sequences (κB sites), initiating the transcription of an array of pro-inflammatory and pro-fibrotic genes (50). The coordinated expression of these molecules amplifies and sustains chronic inflammation and aberrant extracellular matrix remodeling at the ulcer site, while simultaneously inducing fibroblast senescence, impaired keratinocyte migration, and endothelial cell dysfunction (51, 52), collectively contributing to failed tissue repair (as summarized in the ‘Activated AGEs-RAGE axis’ panel of **Figure 3**) and persistent non-healing ulcers.

#### 3.2.3 AMPK signaling pathway

The Adenosine 5’-monophosphate (AMP)-activated protein kinase (AMPK) signaling pathway is centrally involved in glycolipid metabolism and autophagy activation (53, 54). AMPK consists of a catalytic α subunit and regulatory β and γ subunits (55), responding to increases in the cellular AMP/ATP ratio by phosphorylating a wide range of downstream substrates to restore energy balance. AMPK is directly activated when cells experience glucose deprivation, hypoxia, etc., leading to an elevated AMP/ATP ratio. However, in the DU pathological environment, the activation of this pathway is severely compromised. When tissue resides long-term in a metabolically toxic high-glucose environment, AMPK activation was found to significantly increase NCOA4, reversing fibroblast insensitivity to ferroptosis and promoting diabetic wound healing (56). AMPK activation could be a promising therapeutic approach for non-healing diabetic wounds and is closely related to ferroptosis. Once AMPK function is inhibited, its crucial downstream catabolic and homeostatic pathways are consequently weakened. This leads to the obstruction of cellular processes essential for maintaining metabolic homeostasis:

In the DU pathological environment, AMPK signaling is often suppressed. This loss of AMPK activity disrupts crucial catabolic processes, leading to impaired glucose utilization, reduced fatty acid β-oxidation, and enhanced lipogenesis. The synergistic effect of these downstream disruptions is a profound loss of cellular energy homeostasis and increased oxidative stress, severely compromising the metabolic capacity required for healing (57, 58).

The synergistic dysregulation of these downstream effects collectively leads to loss of cellular energy activity, exacerbated oxidative stress, and impaired mechanisms, thereby severely disrupting the metabolic support and microenvironmental stability necessary for DU healing(see the ‘Suppressed AMPK signaling’ pathway in **Figure 3**).

#### 3.2.4 Lipotoxicity and associated pathways

Lipotoxicity is a core pathological issue in DUs, fundamentally involving direct cellular damage caused by lipid overload. This process is not a passive accumulation but actively disrupts normal cell function and survival by activating specific signaling pathways, with the generation and action of harmful lipid species guiding disease progression.

Diabetes and its complications are driven by lipotoxicity, characterized by abnormal lipid accumulation in non-adipose tissues. Among the most dangerous sphingolipids are those promoting cell death, insulin resistance, and reduced insulin gene expression (59). A key component of sphingolipid metabolism is ceramide. Ceramide acts as a critical toxic lipid second messenger. It directly induces insulin resistance and impairs mitochondrial function, the latter triggering massive ROS production. Furthermore, ceramide potently induces apoptosis, thereby directly eliminating crucial reparative cells (60). Increased intracellular free fatty acids (FFAs) also lead to diacylglycerol (DAG) accumulation. DAG activates classical protein kinase C (PKC) family members, which phosphorylate specific serine sites on insulin receptor substrates (IRS) (61), a key mechanism interrupting insulin signaling and exacerbating local insulin resistance. The lipotoxic microenvironment also profoundly influences immune responses. High levels of FFAs and oxidized lipids promote the polarization of tissue macrophages towards a pro-inflammatory M1 phenotype. These activated M1 macrophages then secrete abundant inflammatory cytokines (e.g., TNF-α, IL-1β, IL-6) (62), thereby sustaining and amplifying chronic inflammation in the ulcer area and directly disrupting healing.

There is a profound molecular convergence between the lipotoxic environment and ferroptosis. Acyl-CoA Synthetase Long-Chain Family Member 4 (ACSL4) is the central hub linking the two. Polyunsaturated fatty acids (PUFAs) like arachidonic acid (AA) and adrenic acid (Ada) are highly susceptible to lipid peroxidation. Free PUFAs can serve as substrates for lipid signaling mediators, but they need to be esterified into membrane phospholipids and oxidized to transmit the ferroptosis signal (refer to the ‘Lipotoxicity’ section in **Figure 3**). ACSL4 and lysophosphatidylcholine acyltransferase 3 (LPCAT3) are key enzymes in the biosynthesis and remodeling of phosphatidylethanolamine (PE). Therefore, inhibiting the expression of ACSL4 and LPCAT3 can reduce the accumulation of peroxidizable lipid substrates, thereby suppressing ferroptosis (63). This implies that in DUs, lipotoxicity not only causes damage through traditional pathways but also profoundly enhances cellular susceptibility to ferroptosis by providing abundant, readily peroxidizable membrane phospholipids. Thus, ACSL4 serves as a crucial molecular bridge between GLMDs and ferroptosis, providing a theoretical basis for subsequent targeted therapies against ferroptosis. This direct link exemplifies how the lipotoxic microenvironment of a DU does not merely cause generic cellular stress but actively rewires lipid metabolism to dramatically increase the abundance of phospholipid substrates that are exquisitely sensitive to peroxidation, thereby setting the stage for ferroptosis execution.

## 4 Interaction between ferroptosis and glycolipid metabolism in diabetic ulcers

### 4.1 Ferroptosis counteracts metabolism and the microenvironment

**Figure 4:**
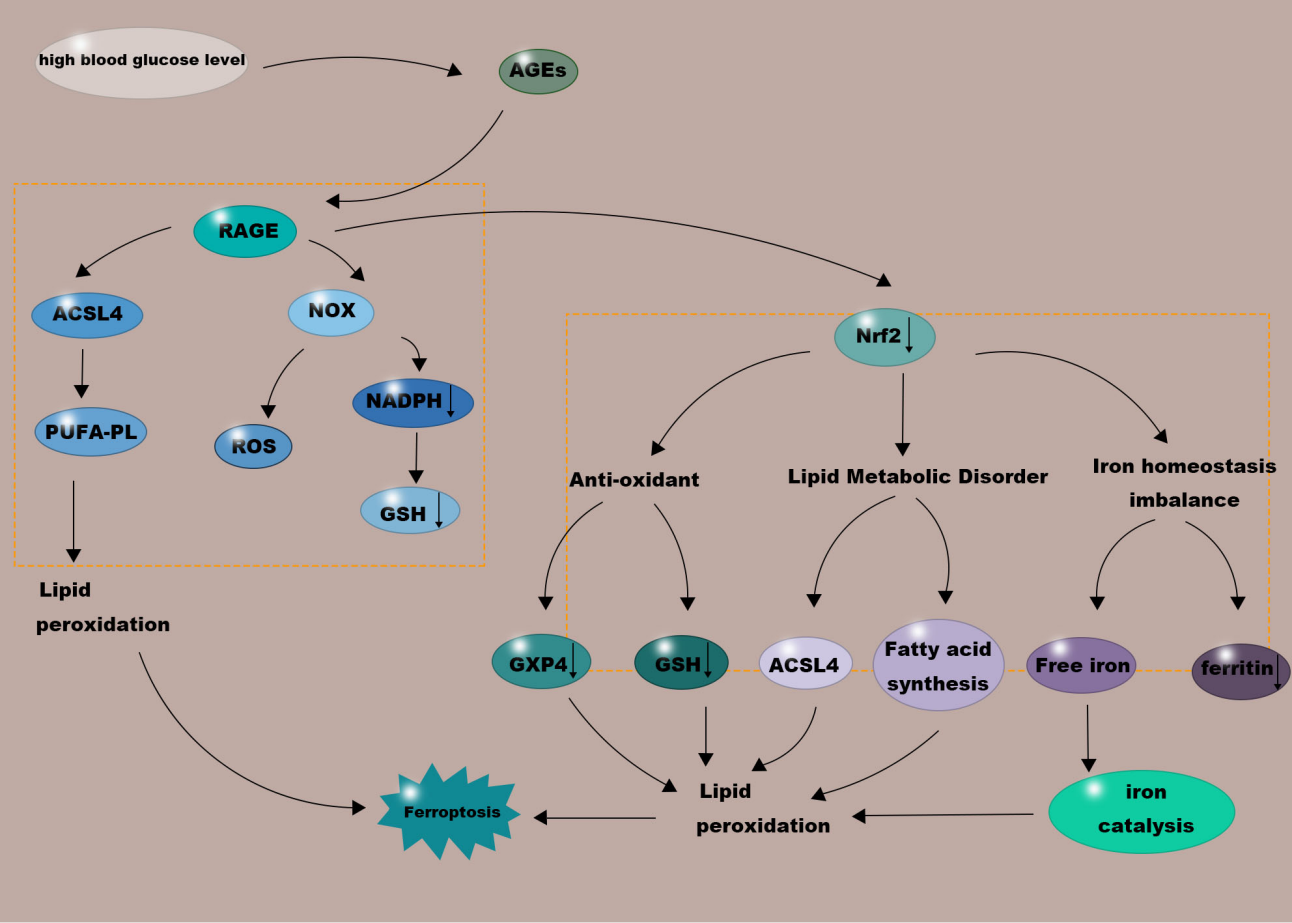
The vicious cycle linking glycolipid metabolic disorders and ferroptosis in diabetic ulcers. This model illustrates the self-sustaining feedback loop that perpetuates tissue damage and impairs healing. The cycle progresses through four key phases: (1) GLMDs Initiate Stress: Hyperglycemia and lipotoxicity create a metabolically toxic microenvironment. This upregulates ACSL4 (increasing peroxidizable lipid substrates), disrupts iron homeostasis (leading to iron overload and Fenton reaction), and suppresses the Nrf2 pathway (weakening antioxidant defenses via GPX4). (2) Ferroptosis Executes Cell Death: The confluence of abundant lipid substrates, catalytic iron, and impaired defenses leads to lethal lipid peroxidation, triggering ferroptotic death of reparative cells (e.g., endothelial cells, fibroblasts). (3) Inflammation Amplifies Damage: Dying cells release damage-associated molecular patterns (DAMPs, e.g., HMGB1, ATP). These activate immune cells (e.g., macrophages), fueling NF-κB/NLRP3 inflammasome signaling and the secretion of potent pro-inflammatory cytokines (e.g., TNF-α, IL-1β). (4) Metabolism Worsens: Inflammatory mediators, particularly TNF-α, exacerbate local insulin resistance and upregulate lipogenic pathways (including ACSL4), thereby reinforcing the initial glucolipid metabolic disorders. Key molecular hubs (e.g., ACSL4, Nrf2, AGEs-RAGE axis) critically connect the metabolic dysfunction to the cell death process. Breaking this cycle at any node represents a promising therapeutic strategy.

Ferroptosis is not an isolated cell death event. The release of Damage-Associated Molecular Patterns (DAMPs) from ferroptotic cells triggers a potent immune-inflammatory response, which in turn acts upon the ulcer microenvironment, establishing a vicious cycle that impedes healing (see the ‘Feedback Aggravation’ loop in the right half of **Figure 4**). As summarized in **Figure 4** and detailed in its caption, this interaction forms a self-amplifying vicious cycle that progresses from metabolic disorders to ferroptotic cell death, then to inflammatory amplification, and finally back to worsened metabolism, thereby providing a comprehensive mechanistic framework for the refractory nature of DUs.

A characteristic event of ferroptosis—plasma membrane rupture—leads to the release of intracellular contents like ATP and High Mobility Group Box 1 (HMGB1) as DAMPs into the extracellular space (64, 65). These DAMPs act as “danger signals,” recognized by pattern recognition receptors on immune cells such as macrophages, subsequently activating downstream NF-κB and NLRP3 inflammasome signaling pathways. This results in caspase-1 activation and promotes the secretion of large quantities of potent pro-inflammatory cytokines like mature IL-1β and IL-18 (66).

The resulting chronic inflammatory microenvironment profoundly negatively impacts glycolipid metabolism. For instance, TNF-α, a key mediator of insulin resistance in obesity and diabetes, exacerbates insulin resistance in surrounding reparative cells by reducing the tyrosine kinase activity of the IR (67, 68). Concurrently, inflammatory signals can also upregulate ACSL4 expression and promote lipolysis, supplying more lipid substrates for ferroptosis (69). This interaction establishes a self-reinforcing vicious cycle: ‘GLMDs → Ferroptosis → Inflammation → Worsened GLMDs.’ This cycle makes it extremely difficult for DUs to break the pathological impasse and enter the productive repair phase.

### 4.2 Key pathways in the interaction

In DUs, GLMDs drive ferroptosis by simultaneously operating through three synergistic pathways: (i) supplying abundant peroxidizable lipid substrates (primarily via upregulation of ACSL4 activity) (70), (ii) providing catalytic ferrous iron through iron overload that fuels the Fenton reaction (71), and (iii) dismantling key cellular antioxidant defenses, notably by depleting the GSH/GPX4 system and suppressing the Nrf2 pathway (72, 73). The following key molecular hubs critically mediate these effects, translating upstream metabolic disturbances into the execution of ferroptosis. To synthesize these interactions, we highlight several core molecular hubs in **Table 2**, detailing how glycolipid metabolism regulates them and their consequent impact on ferroptosis within the DU vicious cycle.

**Table 2:** Key molecular hubs integrating glycolipid metabolic disorders with ferroptosis in diabetic ulcers.

Molecular hub	Regulation by glycolipid metabolism	Impact on ferroptosis	Role in the DU vicious cycle	Therapeutic potential
ACSL4	Upregulated by high glucose, AGEs-RAGE axis, inflammatory cytokines (e.g., TNF-α)	Activates/Promotes	Central hub linking lipotoxicity to ferroptosis susceptibility	Inhibitors (e.g., Troglitazone) may reduce harmful lipid substrates
Nrf2	Suppressed (“Nrf2 dysfunction”) by high glucose and oxidative stress	Inhibits	Key node for antioxidant defense collapse, paving the way for ferroptosis	Activators (e.g., Orientin) may restore cellular defenses (28)
AGEs-RAGE Axis	Activated by persistent hyperglycemia leading to AGEs accumulation	Promotes	Directly links hyperglycemia to ferroptosis drivers (ACSL4, ROS)	Blocking this axis may simultaneously improve metabolism and inhibit ferroptosis
Insulin/PI3K-Akt Signaling	Disrupted due to insulin resistance	Context-dependent	Upstream initiator, creating a metabolically toxic microenvironment	Improving insulin sensitivity may alleviate metabolic pressure from the source

ACSL4, Acyl-CoA synthetase long-chain family member 4; AGEs, Advanced glycation end products; Nrf2, Nuclear factor erythroid 2–related factor 2; RAGE, Receptor for AGEs; ROS, Reactive oxygen species; TNF-α, Tumor necrosis factor-alpha.

#### 4.2.1 Role of the AGEs-RAGE axis in ferroptosis and glycolipid metabolism

The AGEs-RAGE axis is a core pathway directly linking hyperglycemia to ferroptosis. In DUs, persistent hyperglycemia leads to abundant AGE formation and accumulation in skin tissues. These AGEs act as pathogenic ligands, binding to RAGE on cell membranes and triggering downstream signaling events (21, 74).

AGEs-RAGE axis activation promotes ferroptosis through multiple mechanisms. First, it directly upregulates ACSL4 expression (75). ACSL4 upregulation enhances the cell’s capacity to incorporate PUFAs into membrane phospholipids, thereby increasing the lipid substrate pool for ferroptosis (76, 77). Secondly, AGEs-RAGE signaling potently activates NADPH oxidases (NOX), leading to a burst of ROS generation (78). This not only directly exacerbates lipid peroxidation but also consumes substantial amounts of NADPH. NADPH is a key cofactor for maintaining GSH in its reduced state; its depletion directly leads to decreased GSH levels and impaired GPX4 activity, disrupting a key cellular defense system against ferroptosis (this multi-faceted promotion is integrated within the ‘AGEs-RAGE Axis’ hub in **Figure 4**).

#### 4.2.2 Role of Nrf2 signaling in ferroptosis and glycolipid metabolism

The Nrf2 signaling pathway is a central regulator of the cellular antioxidant stress response. It plays a crucial defensive role in maintaining intracellular redox homeostasis, metabolic balance, and resisting ferroptosis. Under physiological conditions, Nrf2 is bound to its cytosolic inhibitor Keap1 and is maintained in an inactive state subject to rapid ubiquitination and degradation. Upon stimulation by electrophiles or oxidative stress, Keap1 undergoes conformational changes, releasing Nrf2. Free Nrf2 translocates into the nucleus, forms a heterodimer with Maf proteins, and binds to the Antioxidant Response Element (ARE), initiating the transcription of a battery of cytoprotective genes (79).

However, in the DU pathological environment, the activation of this pathway is severely suppressed. Pharmacological activation of Nrf2 can alleviate oxidative stress and accelerate wound healing (80). The impaired Nrf2 function, coupled with sustained metabolic insults, creates a persistently pro-oxidant and pro-inflammatory microenvironment, a state often referred to as “Nrf2 dysfunction.”

The inhibition of Nrf2 significantly enhances cellular susceptibility to ferroptosis through multiple key mechanisms:

GPX4 is the ultimate executor against ferroptosis, and glutathione (GSH) is its essential cofactor. Nrf2 is a master transcription factor regulating the expression of key enzymes for GSH synthesis (e.g., GCLM, GCLC) and the cystine transporter xCT (SLC7A11) (73, 81). When Nrf2 is suppressed, the cell’s ability to synthesize GSH and uptake cystine declines, leading to loss of GPX4 activity, inability to clear lipid peroxides, and directly flipping the “switch” for ferroptosis.

Nrf2 has an important regulatory role in lipid metabolism. Impaired Nrf2 function is associated with upregulated expression of lipid metabolism-related genes like ACSL4, further increasing the susceptibility of membrane phospholipids to peroxidation (82), and potentially making fatty acid synthesis pathways more active, providing richer substrates for lipid peroxidation. Nrf2 participates in maintaining intracellular iron homeostasis by regulating genes such as ferritin heavy chain (FTH) and heme oxygenase-1 (HO-1) (83). Nrf2 dysfunction may lead to aberrant expression of iron storage proteins and increased release of free iron, exacerbating the catalysis of lipid peroxidation (84) (**Figure 4**).

Therefore, in DUs, oxidative stress induced by GLMDs leads to the suppression of the Nrf2 pathway; and the inactivation of Nrf2, in turn, paves the way for ferroptosis by dismantling antioxidant defenses, exacerbating lipid disorders, and disrupting iron homeostasis (positioning Nrf2 as a key dysfunctional node in the cycle shown in **Figure 4**). This positions the Nrf2 pathway as a crucial core molecular node connecting upstream metabolic etiology with downstream cell death events.

## 5 Clinical potential and significance of targeting ferroptosis in diabetic ulcers therapy

Ferroptosis has garnered significant attention in the field of DU therapy. This makes targeting ferroptosis an emerging therapeutic strategy, particularly in the context of glycolipid metabolism involvement. In DUs, targeting ferroptosis offers a promising approach to overcome therapeutic resistance and improve treatment efficacy by modulating iron metabolism pathways. Furthermore, the interplay between ferroptosis and glycolipid metabolism underscores the therapeutic potential of targeting ferroptosis. GLMDs are a hallmark of DU progression and have been shown to regulate key ferroptosis targets. By modulating glycolipid metabolic signaling pathways, it might be possible to increase sensitivity to ferroptosis-inducing therapies, thereby enhancing their effectiveness.

### 5.1 Dual potential of ferroptosis inducers and inhibitors in DU therapy

The pathological complexity of DUs dictates the dual nature of ferroptosis modulation: selectively inducing ferroptosis in dysfunctional, hindering cells while robustly protecting critical reparative cells from ferroptosis.

#### 5.1.1 Therapeutic potential of ferroptosis inducers

Targeting the System Xc^−^–GSH axis aims to deplete intracellular GSH, the primary antioxidant, thereby lifting the inhibition on lipid peroxidation. Erastin and its derivatives effectively inhibit System Xc^−^ activity. In the DU environment, senescent fibroblasts, being under high oxidative stress, exhibit immunity to ferroptosis induced by high glucose. Application of Erastin can effectively clear senescent fibroblasts from DU wounds, accelerating wound healing (16).

GPX4, being the core defense line against ferroptosis, is another important target. Small molecules like RSL3 and STING agonists can directly and irreversibly inhibit GPX4 activity, triggering intense lipid peroxidation and cell death (17, 85). However, due to their potent effects and narrow therapeutic window, systemic application risks damaging normal tissues. Future directions involve developing targeted delivery systems, such as hydrogels loaded with compounds like 4-Octyl itaconate (4OI). These systems aim to achieve the precise elimination of hindering cells, which would significantly improve therapeutic efficacy and reduce side effects (86).

#### 5.1.2 Protective strategies with ferroptosis inhibitors

Tissue iron overload in diabetes is a key driver of ferroptosis. Studies confirm that hydrogels loaded with the iron chelator Deferoxamine (DFO) significantly improve wound healing in DU animal models. The mechanism involves not only inhibiting ferroptosis in reparative cells but also stabilizing Hypoxia-Inducible Factor-1α (HIF-1α), leading to upregulated expression of Vascular Endothelial Growth Factor (VEGF) and promoting angiogenesis (18). DFO can be administered topically, effectively chelating excess free iron from the ulcer bed, suppressing the Fenton reaction at its source.

Ferrostatin-1 (Fer-1) and Liproxstatin-1 (Lip-1) are currently the most potent specific ferroptosis inhibitors. They act as radical-trapping antioxidants, directly integrating into cell membranes and interrupting the chain propagation of lipid peroxidation (87). In streptozotocin (STZ)-induced diabetic mouse ulcer models, topical application of Fer-1 or Lip-1 significantly protected endothelial cells and fibroblasts, reduced tissue damage, and promoted granulation tissue formation and re-epithelialization, demonstrating the direct effectiveness of inhibiting ferroptosis in DU treatment (17, 88).

### 5.2 Targeting glycolipid metabolism to regulate ferroptosis

Given that GLMDs are upstream events driving ferroptosis in DUs, intervening in glycolipid metabolism represents a strategy to fundamentally regulate cell fate.

The classic antidiabetic drug Metformin, besides controlling blood glucose, can activate the AMPK pathway, improve mitochondrial function, and indirectly enhance cellular antioxidant capacity, thereby conferring resistance to ferroptosis in reparative cells (89). SGLT2 inhibitors (e.g., Empagliflozin) may prevent the development of ferroptosis in diabetic kidney disease potentially by promoting AMPK-mediated NRF2 activation, altering systemic and local energy substrate utilization (90). Furthermore, SGLT2 inhibitors promote revascularization in diabetic mouse hindlimb ischemia by inhibiting ferroptosis (91), although their specific role in DUs requires further investigation. PPARα agonists promote fatty acid β-oxidation, reduce intracellular lipid accumulation, thereby lowering the risk of lipid peroxidation at the substrate level (92). Inhibiting key enzymes like ACSL4 can reduce the generation of readily peroxidized phospholipids, representing another effective strategy for preventing ferroptosis at the terminal of lipid metabolism. Representative agents for each strategy, their mechanisms, and preclinical evidence in DU models are listed in **Table 3**. The clinical feasibility of repurposing existing drugs is further evaluated in **Table 4**, which assesses their potential effects on DU healing and links to ferroptosis regulation based on current evidence.

**Table 3:** Representative therapeutic strategies targeting the ferroptosis-glucolipid metabolism axis in diabetic ulcers.

Strategy type	Representative agent	Primary target	Mechanism of action	Potential application/evidence in DU
Ferroptosis Inducers	Erastin	System Xc^−^	Depletes GSH, disinhibiting lipid peroxidation	Clears senescent fibroblasts in the wound bed, accelerating healing (16)
	RSL3, ML162	GPX4	Directly inhibits GPX4 activity, leading to lipid peroxide accumulation	Induces death of obstructive cells; narrow therapeutic window requires targeted delivery (17)
Ferroptosis Inhibitors	Deferoxamine (DFO)	Fe^2+^	Chelates free iron, suppressing the Fenton reaction	DFO-loaded hydrogel dressings improve healing in animal models and promote angiogenesis (18)
	Ferrostatin-1 (Fer-1)	Lipid radicals	Traps lipid radicals, halting the peroxidation chain reaction	Protects endothelial cells and fibroblasts, promoting ulcer healing in diabetic mice (17, 88)
Metabolism-Targeting Agents	Metformin	AMPK	Activates AMPK, indirectly enhancing antioxidant capacity	Confers ferroptosis resistance to reparative cells (89)
	SGLT2 Inhibitors	SGLT2	Putatively via the AMPK/NRF2 pathway	Shows anti-ferroptosis potential in diabetic nephropathy and hindlimb ischemia models (90, 91)
	PPARα Agonists	PPARα	Promotes fatty acid β-oxidation, reducing lipid accumulation	Reduces generation of peroxidation-sensitive phospholipids, preventing ferroptosis at the substrate level (92)

AMPK, AMP-activated protein kinase; DFO, Deferoxamine; DU, Diabetic ulcer; GSH, Glutathione; NRF2, Nuclear factor erythroid 2–related factor 2; SGLT2, Sodium-glucose cotransporter 2.

**Table 4:** Potential implications of commonly used anti-diabetic agents and ferroptosis modulators for diabetic ulcer therapy.

Drug class	Representative agent (s)	Primary metabolic action	Potential/known effects on DU	Proposed link to ferroptosis (Mechanism)	Level of evidence (DU context)
Biguanides	Metformin	Activates AMPK, improves insulin sensitivity, suppresses hepatic gluconeogenesis.	May improve healing outcomes clinically; benefits attributed to improved microcirculation and anti-inflammatory effects.	Inhibition: Putatively via AMPK activation, ameliorating mitochondrial dysfunction and oxidative stress, thereby enhancing cellular resilience (93, 94).	Preclinical/Indirect clinical
SGLT2 Inhibitors	Empagliflozin, Dapagliflozin	Promotes urinary glucose excretion, lowers blood glucose, body weight; cardio-renal benefits.	May reduce limb amputation risk (data debated); beneficial hemodynamic and metabolic effects are theorized to aid healing.	Inhibition: Shown to attenuate ferroptosis in diabetic kidney and hindlimb ischemia models via the AMPK/NRF2 pathway (90, 91).	Preclinical (strong); Indirect clinical
GLP-1 Receptor Agonists	Liraglutide, Semaglutide	Glucose-dependent insulin secretion, glucagon suppression, delayed gastric emptying, weight loss.	Anti-inflammatory and endothelial-improving properties are theoretically beneficial; direct DU trial data are limited.	Potential Inhibition: Likely indirect via systemic reduction of inflammation and oxidative stress; precise ferroptosis-related mechanisms require elucidation.	Preclinical (anti-inflammatory); Limited direct DU evidence
Ferroptosis Inhibitors	Deferoxamine (DFO), Ferrostatin-1 (Fer-1), Liproxstatin-1 (Lip-1)	Not hypoglycemics. DFO: Iron chelator; Fer-1/Lip-1: Radical-trapping antioxidants.	Direct Evidence: Topical application in animal DU models (18) significantly reduces tissue damage, promotes angiogenesis and healing (17, 88)	Direct Inhibition: DFO chelates labile iron to suppress the Fenton reaction; Fer-1/Lip-1 halts lipid peroxidation chain propagation (17, 87, 88).	Strong preclinical (*in vivo* DU models)

AMPK, AMP-activated protein kinase; DU, Diabetic ulcer; GLP-1, Glucagon-like peptide-1; NRF2, Nuclear factor erythroid 2–related factor 2; SGLT2, Sodium-glucose cotransporter 2.

### 5.3 Clinical feasibility of repurposing metabolic drugs

Despite the robust preclinical rationale for targeting the ferroptosis-GLMD axis, a critical assessment of clinical feasibility is warranted, particularly regarding the repurposing of established anti-diabetic agents. Currently, direct evidence from randomized controlled trials (RCTs) demonstrating the anti-ferroptotic efficacy of these drugs in DU patients is lacking. However, accumulating preclinical and indirect clinical data provide a strong foundation for future targeted investigations.

Metformin, a first-line therapy, has been shown in preclinical models to activate AMPK, improve mitochondrial function, and alleviate oxidative stress, thereby conferring resistance to ferroptosis in various cell types (95). While no RCT has directly linked its DU healing benefits to ferroptosis inhibition, its pleiotropic effects on cellular metabolism and redox homeostasis align closely with the proposed mechanistic axis.

SGLT2 inhibitors (e.g., Empagliflozin) have demonstrated cardio-renal protective effects in large-scale clinical trials. Preclinical studies suggest these benefits may be partly mediated through the AMPK/NRF2 pathway, reducing ferroptosis in models of diabetic kidney disease and hindlimb ischemia (90, 91). Although dedicated DU trials are needed, *post-hoc* analyses of cardiovascular outcome trials indicate potential benefits on limb outcomes, offering a compelling rationale for specifically studying their impact on ferroptosis in DU wounds.

PPARα agonists (e.g., Pioglitazone) improve systemic lipid metabolism and promote fatty acid β-oxidation, theoretically reducing the lipid substrate pool available for peroxidation (92). Their clinical use in diabetes is established, but like the others, their direct anti-ferroptotic effect in human DU tissue remains an open question.

In summary, while these metabolic modulators lack conclusive, indication-specific clinical proof for ferroptosis inhibition in DUs, their excellent safety profiles, widespread use, and congruent mechanisms of action make them ideal candidates for drug repurposing strategies. Future clinical studies incorporating ferroptosis-specific biomarkers are essential to validate this hypothesis and translate the promising preclinical axis into tangible patient benefits.

### 5.4 Clinical translation challenges and future perspectives

Despite promising preclinical results, translating ferroptosis-targeted therapies for DUs to the clinic faces several challenges. Precisely distinguishing and targeting “harmful” versus “beneficial” cell populations is the core obstacle for the clinical application of ferroptosis inducers. This relies on precise mapping of ferroptosis susceptibility across different cell types in DUs at the single-cell level. There is a current lack of reliable biomarkers for the rapid clinical assessment of ferroptosis levels in ulcer tissue. Identifying detectable indicators in tissue fluid or wound exudate, such as lipid peroxidation derivatives (MDA, 4-HNE), redox status, or iron metabolism-related proteins, is crucial for patient stratification, efficacy evaluation, and prognosis determination, enabling personalized ferroptosis modulation regimens.

## 6 Conclusions and perspectives

The intricate crosstalk between ferroptosis and GLMDs forms a self-amplifying vicious cycle that is central to the pathogenesis and non-healing nature of DUs. In this cycle, GLMDs fuel ferroptosis by providing lipid substrates, catalytic iron, and a pro-oxidant milieu, while ferroptotic cell death, in turn, exacerbates local inflammation and metabolic dysfunction. This bidirectional interplay establishes a pathogenic axis, with molecular hubs such as ACSL4, Nrf2, and the AGEs-RAGE pathway serving as critical connectors and promising therapeutic targets.

To translate this mechanistic understanding into clinical breakthroughs, future research must prioritize several key directions:

Elucidating Cell-Type Specificity: Utilizing single-cell RNA sequencing and spatial transcriptomics to map the susceptibility and functional impact of ferroptosis across diverse cell populations (e.g., fibroblasts, keratinocytes, endothelial cells, macrophages) within DU tissues. This will inform strategies for selective modulation.

Discovering Translational Biomarkers: Identifying and validating reliable, minimally invasive biomarkers of ferroptosis activity in DU patients (e.g., specific lipid peroxidation products in wound exudate or serum). Such biomarkers are crucial for patient stratification, monitoring therapy response, and guiding personalized treatment regimens.

Developing Targeted Delivery Systems: Engineering advanced biomaterials (e.g., responsive hydrogels, nanoparticle-based carriers) for the spatiotemporally controlled and cell-selective delivery of ferroptosis modulators (inducers or inhibitors) to the ulcer site, thereby maximizing efficacy and minimizing systemic side effects.

Repurposing and Combining Therapies: Systematically evaluating the anti-ferroptotic effects of existing metabolic drugs (e.g., SGLT2 inhibitors) in preclinical DU models and prospective clinical cohorts. Furthermore, rational combination therapies that simultaneously target the metabolic (e.g., insulin sensitizers) and ferroptotic (e.g., iron chelators) arms of the axis should be explored for synergistic potential.

Looking forward, combining ferroptosis modulation with emerging regenerative strategies, such as stem cell therapy, represents a promising frontier. Recent perspectives suggest that targeting programmed cell death can significantly enhance the survival and efficacy of stem cells, offering novel avenues for treating diabetes and its complications (96).

By bridging these knowledge and technological gaps, targeting the ferroptosis-GLMD axis holds immense promise for evolving from a compelling pathogenic concept into a revolutionary therapeutic strategy, ultimately improving the prognosis and quality of life for patients suffering from diabetic ulcers.
